# Clinical Impact and Cost-Effectiveness of Whole Exome Sequencing as a Diagnostic Tool: A Pediatric Center’s Experience

**DOI:** 10.3389/fped.2015.00067

**Published:** 2015-08-03

**Authors:** C. Alexander Valencia, Ammar Husami, Jennifer Holle, Judith A. Johnson, Yaping Qian, Abhinav Mathur, Chao Wei, Subba Rao Indugula, Fanggeng Zou, Haiying Meng, Lijun Wang, Xia Li, Rachel Fisher, Tony Tan, Amber Hogart Begtrup, Kathleen Collins, Katie A. Wusik, Derek Neilson, Thomas Burrow, Elizabeth Schorry, Robert Hopkin, Mehdi Keddache, John Barker Harley, Kenneth M. Kaufman, Kejian Zhang

**Affiliations:** ^1^Division of Human Genetics, Cincinnati Children’s Hospital Medical Center and Department of Pediatrics, University of Cincinnati College of Medicine, Cincinnati, OH, USA; ^2^Myriad Genetics Laboratories, Inc., Salt Lake City, UT, USA; ^3^Center for Autoimmune Genomics and Etiology (CAGE), Cincinnati Children’s Hospital Medical Center and Department of Pediatrics, University of Cincinnati College of Medicine, Cincinnati, OH, USA; ^4^US Department of Veterans Affairs Medical Center, Cincinnati, OH, USA

**Keywords:** whole exome sequencing, next generation sequencing, diagnosis, children, clinical utility, pediatrics

## Abstract

**Background:**

There are limited reports of the use of whole exome sequencing (WES) as a clinical diagnostic tool. Moreover, there are no reports addressing the cost burden associated with genetic tests performed prior to WES.

**Objective:**

We demonstrate the performance characteristics of WES in a pediatric setting by describing our patient cohort, calculating the diagnostic yield, and detailing the patients for whom clinical management was altered. Moreover, we examined the potential cost-effectiveness of WES by examining the cost burden of diagnostic workups.

**Methods:**

To determine the clinical utility of our hospital’s clinical WES, we performed a retrospective review of the first 40 cases. We utilized dual bioinformatics analyses pipelines based on commercially available software and in-house tools.

**Results:**

Of the first 40 clinical cases, we identified genetic defects in 12 (30%) patients, of which 47% of the mutations were previously unreported in the literature. Among the 12 patients with positive findings, seven have autosomal dominant disease and five have autosomal recessive disease. Ninety percent of the cohort opted to receive secondary findings and of those, secondary medical actionable results were returned in three cases. Among these positive cases, there are a number of novel mutations that are being reported here. The diagnostic workup included a significant number of genetic tests with microarray and single-gene sequencing being the most popular tests. Significantly, genetic diagnosis from WES led to altered patient medical management in positive cases.

**Conclusion:**

We demonstrate the clinical utility of WES by establishing the clinical diagnostic rate and its impact on medical management in a large pediatric center. The cost-effectiveness of WES was demonstrated by ending the diagnostic odyssey in positive cases. Also, in some cases it may be most cost-effective to directly perform WES. WES provides a unique glimpse into the complexity of genetic disorders.

## Introduction

Mendelian diseases account for a significant number of pediatric disorders. Recently, a systematic review of the records of 5,747 consecutive admissions in 1996 to Rainbow Babies and Children’s Hospital (Cleveland, OH, USA) found an underlying disorder with a significant genetic component in 71% of admitted children (1). Diagnosing patients with complex phenotypes generally involves physical examination, detailed family history, complementary tests such as radiography and metabolite analysis, and genetic testing. A significant proportion of patients undergo extensive genetic testing including karyotyping, array-based comparative genomic hybridization, Sanger sequencing, and multigene next-generation sequencing panels but still remain undiagnosed (2). Accurate diagnosis potentially benefits patients and their families by altering clinical management, predicting recurrence risks, providing prognosis, and ending the diagnostic odyssey that is invasive, time consuming and costly. Clinical diagnosis is therapeutic in its own right for the patient/family, as a result of ending the “diagnostic odyssey” – quite apart from the tangible clinical benefits.

The advent of next-generation sequencing technologies has provided an opportunity to affordably screen a patient’s entire exome to establish genetic basis of disease (3–9). The “exome” is the component of the genome that predominantly encodes protein; these segments are referred to as “exons” and can include non-coding exons. The exome comprises about 1% of the genome and is, so far, the component most likely to include interpretable mutations that result in clinical phenotypes. Whole exome sequencing (WES) involves determination of the DNA sequence of most of these protein-encoding exons and may include some DNA regions that encode RNA molecules that are not involved in protein synthesis. The utility of WES to identify variants causative of Mendelian disorders has been clearly demonstrated in identifying novel candidate genes for Miller syndrome, Fowler syndrome, Perrault Syndrome, and many other disorders (10–17). However, the clinical utility of exome sequencing in pediatric patients needs further examination (18–22). Herein, we report a series of our first 40 consecutive pediatric cases that were referred for WES in a clinical laboratory. We demonstrate the clinical utility of WES in a pediatric setting by describing our patient cohort, calculating the diagnostic yield, detailing the cases in which clinical management was altered, and potential cost-effectiveness of WES as a single test by examining the number and types of genetic tests that were performed prior to WES that add to the cost of diagnostic workups.

## Patients, Materials, and Methods

### Clinical samples

Forty pediatric patients referred by medical specialists (Medical geneticists 77%, Immunologists 15%, Cardiologists 3%, and others 3%) for exome sequencing have had the analysis and results disclosure completed. The patients in this cohort had diverse clinical features and these are summarized in Table 1. Before referral, all patients had undergone extensive diagnostic evaluations (e.g., aCGH microarray, targeted gene tests/panels, metabolic screening, clinical genetic evaluations, and other laboratory workup) that did not lead to a unifying diagnosis. Consent for clinical WES was obtained from the patients and/or their family. Internal review board (IRB) approval was obtained at Cincinnati Children’s Hospital Medical Center (CCHMC) for this retrospective study.

**Table 1:** **Patient demographic information and detailed description of presenting symptoms**.

Case ID	Gender	Age at presentation (months)	Age at testing (months)	Race/ethnicity	Primary disease classification	Presenting symptoms
1	Male	19	36	Caucasian/other (Japanese)	Mitochondrial disorders	Hypotonia, fatigue, speech apraxia, insomnia, fevers, leukopenia, eosinophilic esophagitis, leg length discrepancy
2	Male	6?	60	Caucasian/native American	Neurological disorders	Dysmorphic facial features, intellectual disability, eczema, abnormal gait, developmental delay, lack of verbal skills, hypotonia, seizures, epilepsy
3	Male	3	24	Other (middle Eastern)	Immunodeficiencies	Autoimmune hemolytic anemia, recurrent immune thrombocytopenic purpura, non-specific immune dysfunction
4	Male	12	24	Caucasian	Immunodeficiencies	Common variable immunodeficiency-like symptoms, pan-hypogammaglobulinemia, alopecia universalis, reduced memory B cells, gastroesophageal reflux disease, left midfoot valgus
5	Male	<5	108	Caucasian	Mitochondrial disorders	Hypotonia, gross motor delay, joint hypermobility, poor growth, eosinophilic esophagitis, osteopenia, cyclic recurrent periodic fevers
6	Male	6	72	Caucasian/native American	Multiple congenital anomalies	Autism, intellectual disability, speech and motor apraxia, global developmental delay, gross and fine motor delays, hypotonia, dysmorphic features including midface hypoplasia, flat profile with deep set eyes, frontal bossing, bilateral fifth finger clinodactyly and tapered fingers
7	Male	Birth	12	Caucasian	Immunodeficiencies and multiple congenital anomalies	Immunodeficiency: multiple infections, T-B + NK + severe combined immunodeficiency with dermatitis and hair loss; congenital anomalies: cervical and lumbar kyphosis, basilar skull anomaly, short stature, bilateral microtia, malar prominence, narrow alae nasi, cupid bow lip, retrognathia, external ear malformation. Other features: hearing loss, undescended testicles, thrombocytopenia
8	Female	24	24	Caucasian	Immunodeficiencies	Acute liver failure due to immune dysregulation of unknown etiology; profound lymphopenia, decreased NK cell function, hepatomegaly, elevated liver enzymes
9	Female	18	192	Caucasian	Mitochondrial disorders	Febrile and tonic/clonic seizures, intellectual disability, developmental regression, chronic constipation, back pain, photophobia, nosebleeds, language regression, infrequent urination
10	Male	<12	144	Caucasian/native American	Neurological disorders and multiple congenital anomalies	Hypotonia, mild intellectual disability, cerebellar hypoplasia, ataxia, global developmental delay, exercise intolerance
11	Female	<12	132	Caucasian/native American	Neurological disorders	Global developmental delay, intellectual disability, developmental regression, autism, macrocephaly, seizures, hypomyelination
12	Male	2	1	Other (Nepalese)	Multiple congenital anomalies	Chronic hepatitis, jaundice, cirrhosis, hepatomegaly, end stage liver disease, global developmental delay, hypotonia, failure to thrive, midface hypoplasia, narrow palate, postural kyphosis, mild left ventricular dilation, mild left ventricular trabeculation
13	Female	1	168	Caucasian	Immunodeficiencies	Atypical common variable immunodeficiency, absent B cells, lymphohematopoietic disorder, hypogammaglobulinemia, chronic lymphocytic hepatitis, recurrent sinopulmonary infections, granulomatous hepatitis causing cirrhosis, portal hypertension
14	Female	6	36	Caucasian	Neurological disorders	Infantile onset dopa-responsive dystonia, gross and fine motor delay, swallowing difficulty
15	Male	6	60	Caucasian/native American	Neurological disorders	Epilepsy, visual impairment, global developmental delay, hypotonia, failure to thrive, clinodactyly, intellectual disability
16	Male	Not available, adopted	384	Caucasian	Multiple congenital anomalies	Progressive optic atrophy, ataxia, moderate sensorineural hearing loss, muscle weakness, vertigo, erythrocytosis, horizontal nystagmus
17	Male	6	24	Caucasian/Ashkenazi Jewish	Neurological disorders	Infantile dystonia, hypertonicity, gross motor delay
18	Male	1.5	72	Caucasian	Immunodeficiencies	Duodenal web, esophageal strictures, intestinal dysmotility, autoamputation, toe necrosis, acrocyanosis, vasculitis, arteritis, necrosis, dysphagia, infections
19	Male	Birth	84	Caucasian	Multiple congenital anomalies	Joint laxity, hypotonia, exercise intolerance, arthralgias, excessive bruising, fatigue, immune dysregulation, chirari malformation, developmental regression
20	Male	<24	156	Caucasian	Multiple congenital anomalies	Microcephaly, apraxia, cognitive impairment, poor weight gain, dysmorphic facial features
21	Male	<24	96	Caucasian	Multiple congenital anomalies	Syndromic heart anomaly: mitral valve stenosis, hypoplastic aortic arch, left ventricular non-compaction, dysmorphic facial features, short stature, developmental delay
22	Female	<7?	168	Caucasian	Immunodeficiencies	Combined immune deficiency of undetermined genetic etiology, recurrent EBV infection, bone marrow transplant, Grave’s disease, cataracts, ptosis and choroidal nevus
23	Male	1	24	Caucasian	Immunodeficiencies and multiple congenital anomalies	Hypogammaglobulinemia, recurrent infections, recurrent fevers, fine motor and speech delay, feeding problems, hypotonia, macrocephaly, prominent forehead, deep set eyes, thin upper lip, long fingers and toes, and persistent fetal fingerpads
24	Female	Birth	60	Caucasian	Neurological disorders	Severe hypotonia, absent swallow, developmental delay, muscle pain, fatigue, ptosis, heat intolerance, hearing loss, hypersecretions, low set ears, and a high arched palate
25	Female	Infant	204	Caucasian	Multiple congenital anomalies	Leukoencephalopathy, global developmental delay, hypotonia, ataxia, cryptogenic partial complex epilepsy, dysphagia, seizures, flattened midface, prognathism, bilaterally cupped ears with simplified anti-helix
26	Male	Birth	48	Caucasian	Multiple congenital anomalies	Skeletal dysplasia with mild metaphyseal flaring, very short long bones with micromelia, bell-shaped thorax, bowing of the tibias, and platyspondyly with secondary lordosis, scoliosis, kyphosis
27	Female	2	24	Caucasian	Neurological disorders	Global developmental delay, hypotonia, infantile onset of seizures, progressive microcephaly, feeding difficulties, gastroesophageal reflux disease, diarrhea, constipation
28	Female	36	36	Caucasian	Immunodeficiencies	Hemophagocytic lymphohistiocytosis, abnormal degranulation of NK cells
29	Male	Birth	96	Caucasian	Multiple congenital anomalies	Encephalopathy, expressive language delay, intellectual disability, and motor impairment, Pierre Robin sequence, hypotonia, undescended testes and dysmorphic facial features
30	Female	Birth	36	Caucasian	Neurological disorders	Agenesis of corpus callosum, polymicrogyria, gray matter heterotropia
31	Male	Birth	72	Caucasian	Mitochondrial disorders	Exercise intolerance, fatigue, hypotonia, ventricular septal defect, patent foramen ovale, eosinophilic esophagitis, severe global developmental delay, difficulty feeding, abdominal pain, heat intolerance, bone lesion in femur, absent speech, intellectual disability, autism, poor balance, hypermobility
32	Male	<5	14	Caucasian/native American	Mitochondrial disorders	Muscle weakness, hypotonia, gastrointestinal dysmotility, gastroesophageal reflux disease, dysphagia, ataxia, epilepsy, autonomic dysfunction, malrotation of small intestine, developmental delay
33	Male	<6	168	Caucasian/other (Chinese/Indian/New Zealander)	Neurological disorders	Fatigue, lactic acidosis, cryptogenic infantile spasms, hypotonia, developmental delay
34	Female	Birth	60	Caucasian	Neurological disorders and multiple congenital anomalies	Mild developmental delay, intractable epilepsy, isolated cataplexy, hypopigmented linear nevi, streaky hypopigmentation on right leg, hypoplastic tissue on right toe, and hand, dysmorphic facial features, shortened right ulna with congenital radial head dislocation
35	Male	<24	96	Caucasian	Mitochondrial disorders	Fatigue, dysmotility, lower extremity spasticity, delayed milestones, acute liver failure, eosinophilic esophagitis, dysruptive behavior disorder, obstructive sleep apnea
36	Female	3	12	Caucasian/other (Filipino, Puerto Rican, Chinese)	Multiple congenital anomalies	Global developmental delays, hypotonia, brachycephaly, upslanting palpebral fissures, broad angulated thumbs, bulbous great toes, clinodactyly
37	Male	Birth	12	Other (Mexican)	Multiple congenital anomalies	Bilateral hearing loss with enlarged vestibular aqueducts; developmental delay, hypotonia
38	Male	66	72	Caucasian	Endocrinology	Atypical type 1 diabetes (lack of ketones), cataract, hepatomegaly
39	Male	Birth	156	Caucasian	Multiple congenital anomalies	Microcephaly, bilateral congenital chorioretinal colobomas, vision loss of left eye, depression, anxiety disorder, intellectual disability
40	Male	<24	60	Caucasian/native American	Neurological disorders and multiple congenital anomalies	Seizures, intermittent weakness, megalocornea, leg movements, poor gross and fine motor skills

*? means the age is not certain*.

### Whole exome sequencing and sanger confirmation

WES and analysis protocols were developed and validated by the CCHMC molecular genetics laboratory of the Division of Human Genetics. Briefly, genomic DNA samples from patients were fragmented by sonication, ligated to Illumina multiplexing paired-end adapters, amplified by means of a polymerase-chain reaction and hybridized to biotin-labeled NimbleGen V3 exome capture reagent (Roche NimbleGen). Hybridization was achieved at 47°C for 64–72 h. After washing and reamplification, paired-end sequencing (2 × 100 bp) was performed on the Illumina HiSeq 2500 platform to provide average sequence coverage of more than 100×, with more than 97% of the target bases having at least 10× coverage. Clinically relevant variants, from proband and parental samples (whenever available), were confirmed by Sanger sequencing.

### Data analysis and annotation

To aid in the clinical interpretation of variants, data were annotated and analyzed using two pipelines: (1) next gene pipeline (NGP) and the (2) genome analysis toolkit (GATK)/golden helix pipeline (GGHP). For NPG, output data from the Illumina HiSeq 2500 were converted from bcl files to FastQ files using the Illumina consensus assessment of sequence and variation (CASAVA) software, version 1.8, and mapped to the reference haploid human-genome sequence (hg19) with NextGene 2.3.4 using default settings. Passing quality control/quality assurance (QC/QA) parameters included >75 million reads, >100× average coverage, >95% at 10× coverage of the target exome, and >200 million unique molecules for each case. Variant calls, which differed from the reference sequence was obtained with NextGene 2.3.4. NextGENe genotyping settings were as follows: non-synonymous variants are generated for CDS ± 20 bp and heterozygous, homozygous and wildtype calls had allele percentages of >20 and <80%, >80 and <20%, respectively. Alamut HT 1.1.8 was used for variant annotation. In-house developed scripts were applied for variant prioritization based on phenotype-genotype correlation (Phenomizer and Pheno2Gene, a CCHMC-developed tool), presence in the literature, inheritance modeling, frequency in exome sequencing project (ESP) and exome aggregation consortium (ExAC) population databases (<1%, unless literature reported with a higher frequency or other lines of evidence supported pathogenicity of variants), mutation type, *in silico* predictions (POLYPHEN, SIFT, and Grantham Scale), and presence of the variant in functional domains. Variants were excluded from the analysis if they had a coverage of <10×.

For GGHP, reads generated by the sequencer were aligned to the human genome with BWA 0.5.9. Then, the aligned reads were checked for quality (QC/QA parameters). Upon passing, the aligned data were preprocessed to reduce sequencing biases using Picard 1.5.3 and GATK Appistry 2013.2. The data were compared to the reference sequence, along with standard control samples and other samples in the batch in order to generate a final list of variants for the exome test using with GATK 7.7.4. The output variant calls were in the standard VCF format. The variants were called with GATK using the following commands: -T Unified Genotyper-dcov 1000-stand_call_conf 30.0-stand_emit_conf 30.0 – min_base_quality_score 20 -A Depth Of Coverage -A Indel Type -A QualByDepth -A ReadPosRankSumTest -A FisherStrand -A MappingQualityRankSumTest -l INFO -glm. The VCF file was analyzed using Golden Helix Software (ver. 7.7.4) as previously reported (23). Briefly, quality filtering to exclude variants was based on alternative allele ratio, genotype quality score value (GQ <20 excluded), and read depth (depth <15× excluded). Genetic filters included keeping variants that had a <1% allele frequency and fit Mendelian inheritance models.

### Data interpretation and reporting

Common and unique variants from both analysis pipelines that remained after filtering were further classified as deleterious mutations, variants of unknown clinical significance (VUCS), or benign variants in the context of their relatedness to the patient’s phenotype, and pathogenic mutations on the American College of Medical Genetics (ACMG) gene list were classified as medically actionable secondary findings. Stringent conditions were employed to identify causative mutations. Specifically, we followed the ACMG guidelines to denote deleterious mutations and binned them into the ACMG category 1 (previously reported to be deleterious) or category 2 (predicted to be deleterious) (24). Previously reported or predicted to be deleterious mutations in genes associated with the patient’s phenotypes were prioritized. Each causative mutation was scrutinized by thorough literature review and database searches. Moreover, the pathogenicity of novel and rare variants was assessed by *in silico* prediction programs. Additionally, patterns of familial segregation were examined for correlation. Moreover, pathogenic variants were identified based on the ACMG secondary finding recommendations (if the family chose to receive this information) (25). At the final stage, clinical exome sequencing data interpretation was performed by a team represented by clinical molecular and medical geneticists, pediatric subspecialists, and genetic counselors. The criteria for a full or partial molecular diagnosis were defined as follows: (1) Full molecular diagnosis – Gene variant(s) that is classified as likely pathogenic or pathogenic explains most or all of the clinical features of the patient. (2) Partial molecular diagnosis – Gene variant(s) that is classified as likely pathogenic or pathogenic explains one or several of the clinical features of the patient.

## Results

### Cohort description

**Figure 1:**
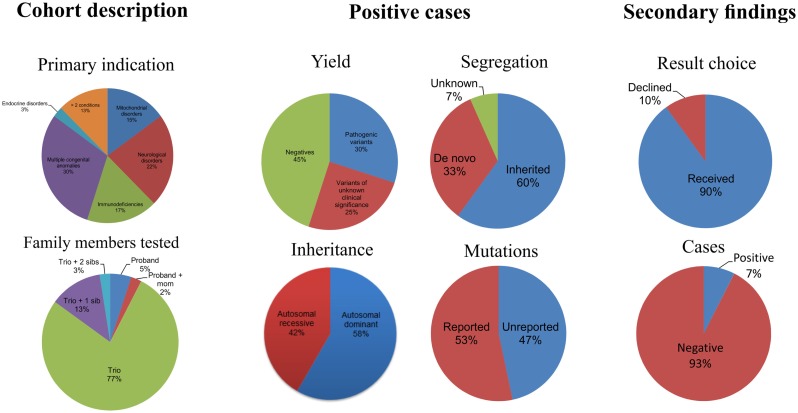
**Descriptive statistics of the patient cohort and the positive exome cases**.

Of the 40 patients, 30, 17, 22, and 25% were patients with primary phenotypes related to multiple congenital anomalies, immunodeficiency, neurological, and mitochondrial disorders, respectively (Table 1; Figure 1, Primary indication). Thirteen percent had clinical features of more than two of the broad aforementioned categories. All patients were under 17 years of age at the time of exome analysis (average age 83.2 months) and much younger at the time of clinical presentation (average age 5.3 months) (Table 1).

### Exome sequencing

As quality control/quality assurance parameters we measured the average coverage, percentage at 10× and 20× per family (Table S1 in Supplementary Material). The typically QC/QA acceptable average coverage was >100× and >95% at 10×.The mean average coverage for the cohort was 125.76×. In addition, the mean percentage coverage at 10× and 20× was 96.85 and 95.40%, respectively. There were several exceptions to the 100× average coverage, namely cases 82, 80, 36, and 38, and after several attempts the average coverage was lower, but other QC/QA parameters including the 95% at 10× were all met. Thus, in rare occasions, exceptions may be made if the sample is limiting, DNA quality is less than optimal, several attempts are made to meet all parameters, or if most of the other QC/QA parameters were met.

On average 32,171 single-nucleotide variants (pre-filtering) and small insertion and deletion, changes were identified in each patient’s exome by comparison with the current reference h19 haploid human genome sequence (Table S1 in Supplementary Material). On average 2031 (post-filtering) of potential clinically useful variants were kept after filters were applied (Table S1 in Supplementary Material). This number has continued to decrease by the utilization of internal normal controls and for current cases it is ~500 variants. Typically, three to eight variants were submitted for Sanger sequencing for each proband and family member(s). More than 84% of the variants selected for potential reporting were confirmed by means of Sanger sequencing. The remaining 16% were proven to be false positives due to unequal allele fractions, poor mapping scores, sequence homology, and insertion and deletion erroneous calls. Within the false positive variants, significant proportions were deletions (16%) and insertions (12%) and the remaining were single nucleotide variants (72%).

### Diagnoses using exome data

Of the 40 probands, 12 carried 18 mutant alleles at 15 different chromosomal loci that satisfied criteria for a full or partial molecular diagnosis (Table 2). The overall rate of a positive molecular diagnosis was 30% (Figure 1, Yield). This group included seven patients with autosomal dominant disease and five with autosomal recessive disease (Figure 1, Inheritance). A diverse group of disorders were represented in the positive patients (Table 2), including primary immunodeficiency, Ehlers–Danlos syndrome and multiple congenital anomalies, such as Otofaciocervical syndrome, Acromesomelic dysplasia Maroteaux type, and Bainbridge-Ropers syndrome.

**Table 2:** **Positive exome cases with pathogenic variants and secondary findings**.

Case ID	Gene(s)	OMIM	Mode of inheritance	Mutation(s)	Frequency (%)		Prediction		Zygosity	Segregation	Literature	Genetic Diagnosis	Altered management
					ESP	ExAC	SIFT	Polyphen	
4	*NFKB2*	*164012*	AD	c.2598_ 2599insT (p.A867fs)	n/a	n/a	–	–	Het	Father: WT Mother: WT *De novo*	(26)	Common variable immunodeficiency 10	(1) End diagnostic odyssey, (2) Start regular endocrine evaluation to monitor potential central adrenal insufficiency, (3) Informative genetic counseling
7	*PAX1*	*615560*	AR	c.463_465del (p.Asn155del)	n/a	n/a	–	–	Hom	Father: Het Mother: Het	This study	Otofaciocervical syndrome 2	(1) End diagnostic odyssey, (2) Informative genetic counseling
11	*NPC1*	*257220*	AR	c.3450C > A (p.N1150K)	n/a	n/a	Deleterious	Probably damaging	Het	Mother: Het	(27)	Niemann-Pick disease, type C1	(1) End diagnostic odyssey, (2) Symptomatic treatment, (3) Prevent complication, (4) Avoid side effects of certain drug/agents, (5) Informative genetic counseling
				c.3019C > G (p.P1007A)	0.02	0.01	Deleterious	Probably damaging	Het	Father: Het	(28)	
16	*GJB2*	*121011*	AD/AR	c.416G > A (p.S139N)	0.08	0.03	Deleterious	Probably damaging	Het	Unknown (Proband only)	(29)	Deafness (DFNB1)	(1) Partial diagnosis established and specific follow-up study recommended, (2) Informative genetic counseling
19	*COL5A1*	*120215*	AD	c.1588G > A (p.G530S)	4.33	3.57	Deleterious	Probably damaging	Het	Mother: Het (clinical information unknown)	(30)	Ehlers-Danlos syndrome	(1) Partial diagnosis established and specific follow up study recommended, (2) Informative genetic counseling
20	*DYRK1A*	*600855*	AD	c.691G > T (p.R231*)	n/a	n/a	–	–	Het	Father: WT Mother: WT *De novo*	This study	Mental retardation, autosomal dominant 7	(1) End diagnostic odyssey, (2) Informative genetic counseling
22	*CTPS1*	*123860*	AR	c. IVS1692-1G > C	0.03	0.01	–	–	Hom	Mother: Het Father: Het Sister: Hom (affected)	(31)	Cytidine-5-prime triphosphate synthetase deficiency1, Immunodeficiency 24	(1) End diagnostic odyssey, (2) Targeted treatment plan, (3) Informative genetic counseling, (4) Secondary finding result in recommended special disease surveillance, (5) Medical concerns for family members
	**MYL2**^c^	*160781*	AD	c.141C > A (p.N47K)	0.06	0.02	Tolerated	Benign	Het	Father: Het (clinical information unknown)	(32)	Familial hypertrophic cardiomyopathy	
23	**FBN1**^c^	*134797*	AD	c.5873G > A (p.C1958Y)	n/a	n/a	Deleterious	Probably damaging	Het	Father: WT Mother: WT *De novo*	(33)	Marfan syndrome	(1) Partial diagnosis established with emerging clinical symptoms, (2) New clinical management plan recommended, (3) Informative genetic counseling
24	*CHRNE*^a^	*100725*	AR	c.422C > T (p.P141L)	n/a	0.00	Deleterious	Probably damaging	Het	Unknown (Proband only)	(34)	Congenital myasthenic syndrome	(1) Established a specific genetic diagnosis, (2) Support a more targeted treatment plan, (3) Informative genetic counseling
				c.529_551del (p.E177Rfs*)	n/a	0.00	–	–	Het		This study	
25	*STXBP1*	*602926*	AD	c.1217G > A (p.H406R)	n/a	n/a	Deleterious	Probably damaging	Het	Father: WT Mother: WT *De novo*	(35)	Early infantile eplileptic encephalopathy, 4	(1) End diagnostic odyssey, (2) Informative genetic counseling
26	*NPR2*^b^	*108961*	AR	c.263A > C (p.K88T)	n/a	n/a	Deleterious	Probably damaging	Hom	Paternal UPD	This study	Acromesomelic dysplasia, Maroteaux type	(1) End diagnostic odyssey, (2) Informative genetic counseling
29	*ASXL3*	*615115*	AD	c.1383_ 1386dupTATC (p.C463Yfs*)	n/a	n/a	–	–	Het	Father: WT Mother: WT *De novo*	This study	Bainbridge-Ropers syndrome	(1) End diagnostic odyssey, (2) Informative genetic counseling
35	**BRCA2**^c^	*600185*	AD	c.3847_ 3848delGT (p.V1283Kfs*)	0.04	0.01	–	–	Het	Father: Het (clinical information unknown)	(36)	Hereditary breast and ovarian cancer syndrome	(1) Achieve partial diagnosis, (2) Disease surveillance suggested, (3) Informative genetic counseling
38	*INS*	*176730*	AD	c.94G > A (p.G32S)	n/a	n/a	Deleterious	Probably damaging	Het	Father: WT Mother: WT *De novo*	(37)	Permanent neonatal diabetes mellitus or type 1b diabetes mellitus	(1) Established specific genetic diagnosis, (2) Complication prevention measurement suggested, (3) Targeted treatment recommended, (4) Informative genetic counseling

*^a^Mutations found to be in *trans* but parents not tested*.

*^b^Uniparental disomy – isodisomy*.

*^c^Secondary findings are bolded*.

*AD, autosomal dominant; AR, autosomal recessive; Het, heterozygous; Hom, homozygous; UPD, uniparental disomy; WT, wildtype; n/a, not available; ESP, exome sequencing project database; ExAC, exome aggregation consortium database*.

*The gene, mutations, mode of inheritance, associated disorders, family segregation, and indicated alteration of medical management are described. Moreover, the frequencies and predictions used, in part, for interpretation of variants are included. Note that all variants were rare with frequencies <1% or have not been reported in ESP and ExAC databases. Note that there was an exception to this rule, *COL5A1* (Case 19), due to the fact that this variant was reported in the literature to be a mutation even at a slightly higher frequency*.

A full range of mutation types were observed in this cohort: three frameshift, two in-frame, two nonsense, two splicing, and eight missense mutations (Table 2). Missense (50%) and frameshift (19%) mutations made up the highest percentages of changes (Figure 1, Mutation type). The majority of these mutations were inherited (58%); however, a significant percentage of *de novo* mutations, defined as mutations present in the proband and not in the parents, were observed (29%) (Figure 1, Segregation). Moreover, 47% of the mutations were previously unreported in the peer-reviewed literature and variant databases (Figure 1, Literature). A total of seven patients had autosomal dominant disorders; of which, three were novel variants that had not been described in the peer-reviewed literature. For the five patients with autosomal recessive disease, parental studies indicated that four had inherited mutant alleles from each carrier parent. The remaining patient, for whom parental samples were not available, was found to have mutations *in trans* by allele-specific PCR.

Variants of unknown clinical significance were found in a number of patients (Table 3). These variants were placed on this table because of pathogenicity prediction inconsistency, higher minor allele frequency, and/or minimal published literature information; however, these genes/variants may explain part of the phenotype. For example, case 6 had two *POLR3B* variants classified as VUCS. c.1958A > T is a rare variant with frequencies less than 1% in ESP and ExAC. On the other hand, c.2218A > G is a variant with a higher frequency, but the predictions are inconsistent with one being probably damaging. Interestingly, both variants supported a compound heterozygous model and the gene is associated with the phenotype described for this patient. Similarly, cases 21, 34, 37, and 39 had higher variant frequencies, but with other lines of evidence that supported their pathogenicity such as a second allele in a compound heterozygous model, predictions that were deleterious and phenotype consistency. Importantly, these cases illustrate the complexity of variant interpretation and that frequency is only one of the variables used to bin them into pathogenicity categories. For cases 11 and 16, in addition to pathogenic variants, compound heterozygous variants classified as VUCS were found in genes which may contribute to the complex phenotypes in these patients. Only VUCSs were observed in cases 1, 2, 3, 6, 21, 23, and 24. These variants may be reclassified in the future once these genes/variants are better understood.

**Table 3:** **Variants of unknown clinical significance**.

Case ID	Gene (s)	Mode of inheritance	Variants(s)	Frequency (%)		Prediction		Zygosity	Segregation	Literature	Disorder
				ESP	ExAC	SIFT	Polyphen	
1	*ACADSB*	AR	c.621G > A (p.W207*)	0.02	0.00	–	–	Het	Father: Het	This study	2-methylbutyryl-CoA dehydrogenase deficiency
									Mother: WT	
			c.454A > T (p.K152*)	0.33	0.21	–	–	Het	Father: WT	This study	
									Mother: Het	
2	*SCN9A*	AD	c.4612T > C (p.W1538R)	0.33	0.21	Deleterious	Benign	Het	Father: Het	(38)	Late-onset chronic non-paroxysmal neuropathic pain
									Mother: WT	
			c.2971G > T (p.V991L)	0.42	3.11	Tolerated	Benign	Het	Father: Het	(39)	Small fiber neuropathy
									Mother: WT	
			c.2794A > C (p.M932L)	0.41	2.95	Deleterious	Benign	Het	Father: Het	(40)	Small fiber neuropathy
									Mother: WT	
3^#^	*CFHR1*	AD/AR	c.19delG (p.7Vfs)	n/a	1.29	–	–	Het	Father: Unknown	This study	Atypical hemolytic uremic syndrome
									Mother: WT	
	*ADAMTS13*	AR	c.686 + 4T > G	n/a	n/a	–	–	Het	Father: Unknown	This study	Thrombotic thrombocytopenic purpura
									Mother: WT	
			c.1852C > G (p.P618A)	8.63	6.31	Deleterious	Probably damaging	Het	Father: Unknown	This study	
									Mother: WT	
	*IL16*	AR?	c.865-3C > T	n/a	n/a	–	–	Het	Father: Unknown	This study	Unknown
									Mother: WT	
			c.2377C > T (p.R793C)	0.01	0.00	Deleterious	Probably damaging	Het	Father: Unknown	This study	
									Mother: Het	
6	*POLR3B*	AR	c.1958A > T (p.D653V)	0.45	0.61	Tolerated	Benign	Het	Father: WT	This study	Hypomyelinating leukodystrophy-8
									Mother: Het	
			c.2218A > G (p.T740A)	6.91	4.70	Tolerated	Probably damaging	Het	Father: Het	This study	
									Mother: WT	
			c.*5G > A	0.03	0.03	–	–	Het	Father: Het	This study	
									Mother: WT	
11	*TDP2*	AR	c.383A > G (p.N128S)	0.00	0.00	n/a	Benign	Het	Father: Het	This study	Intellectual disability, seizures, and ataxia
									Mother: WT	
			c.17G > T (p.C6F)	0.33	0.29	n/a	Benign	Het	Father: WT	This study	
									Mother: Het	
16*	*ITPR1*	AD	c.7568C > T (p.T2523M)	n/a	0.00	Deleterious	Probably damaging	Het	Father: Unknown	This study	Spinocerebellar ataxia types 15 and 29
									Mother: Unknown	
21	*TTN*	AD/AR	c.23117A > G (p.Y7706C)	n/a	n/a	n/a	n/a	Het	Father: WT	This study	Dilated cardiomyopathy, muscular dystrophy, myopathy
									Mother: Het	
			c.851C > T (p.S284L)	n/a	0.01	n/a	n/a	Het	Father: Het	This study	
									Mother: WT	
	*DES*	AD/AR	c.638C > T (p.A213V)	1.37	1.01	Deleterious	Probably damaging	Het	Father: WT	(41)	Dilated cardiomyopathy, limb-girdle muscular dystrophy, myofibrillar myopathy, scapuloperoneal syndrome
									Mother: Het	
	*SMPD1*	AR	c.138_143del	n/a	22.39	–	–	Hom	Father: WT	This study	Niemann–Pick disease type A, B
									Mother: Het	
23	*AIRE*	AR	c.967_979del13 (p.L323fs)	0.15	0.05	–	–	Het	Father: WT	(42)	Autoimmune polyendocrinopathy syndrome type I
									Mother: Het	
24*	*ALMS1*	AR	c.1577_ 1579insCTC (p.P526_ L527insS)	n/a	n/a	–	–	Het	Father: Unknown Mother: Unknown	This study	Alstrom syndrome
			c.69_77del (p.E24_E26del)	n/a	3.84	–	–	Het	Father: Unknown	This study	
									Mother: Unknown	
32	*CACNA1H*	AD?	c.2057C > T (pP686L)	0.11	0.26	Deleterious	Probably damaging	Het	Father: WT	This study	Susceptibility to childhood absence -6 or idiopathic generalized epilepsy -6
									Mother: Het	
34	*RTTN*	AR	c.5060C > G (p.S1687C)	1.68	1.78	Deleterious	Probably damaging	Het	Father: WT	This study	Polymicrogyria with seizures
									Mother: Het	
			c.1665C > A (p.N555K)	1.67	1.69	Deleterious	Probably damaging	Het	Father: WT	This study	
									Mother: Het	
			c.4298G > A (p.R1433Q)	0.05	0.05	Deleterious	Probably damaging	Het	Father: Het	This study	
									Mother: WT	
37	*WFS1*	AD/AR	c.1367G > A (p.R456H)	5.08	5.70	Tolerated	Probably damaging	Hom	Father: Het	(43)	Deafness, autosomal dominant/Wolfram syndrome
									Mother: Het	
			c.1538A > C (p.Y513S)	n/a	0.00	Deleterious	Probably damaging	Het	Father: WT	This study	
									Mother: Het	
	*COL6A3*	AD	c.5833G > C (p.V1945L)	0.02	0.03	Tolerated	Probably damaging	Het	Father: WT	This study	Bethlem myopathy/Ullrich congenital muscular dystrophy
									Mother: Het	
38	*MAPK8IP1*	AD	c.962C > T (p.T321M)	n/a	0.00	Tolerated	Probably damaging	Het	Father: WT	This study	Non-insulin-dependent diabetes mellitus
									Mother: Het	
39	*LRP2*	AR	c.2006G > A (p. 669G > D)	3.52	2.85	Deleterious	Probably damaging	Het	Father: WT	This study	Donnai-Barrow syndrome
									Mother: Het	
			c.4351G > T (p. 1451V > F)	0.30	0.20	Deleterious	Benign	Het	Father: Het	This study	
									Mother: WT	
	*PCNT*		c.4285C > T (p. 1429R > C)	0.79	0.37	Tolerated	Probably damaging	Het	Father: Het	This study	Microcephalic osteodysplastic primordial dwarfism, type II
									Mother: WT	
			c.8401G > C (p. 2801V > L)	0.05	0.01	Tolerated	Probably damaging	Het	Father: WT	This study	
									Mother: Het	
40	*SCN9A*	AD	c.3799C > G (p.L1267V)	0.23	0.14	Deleterious	n/a	Het	Father: WT	(44)	Dravet syndrome/febrile seizures
									Mother: Het	

*^#^Father’s sample is not available for testing*.

*Proband only.

*? means the age is not certain*.

*Het, heterozygous; Hom, homozygous; WT, wildtype; n/a, not available; ESP, exome sequencing project database; ExAC, exome aggregation consortium database*.

*These variants were classified based on the ACMG guidelines. Generally, these variants may partly explain the patient’s phenotype and/or there was not enough evidence to classify them in the “benign” or “pathogenic” categories due to inconsistencies in the pathogenicity prediction program, slightly higher frequency, weak amino acid conservation, segregation, and/or lack of functional data. Note that most variants were unreported or rare (<1%). Note that there were several exceptions to this rule; however, these variants were classified as VUCS because other lines of evidence supported their pathogenicity*.

### Altered management of patients

In positive cases, WES results led to a change in the medical management of the patients (Table 2). Willig et al. defined medical management in a broader sense, namely, usefulness of genomic sequencing, return of results before discharge or death, genetic or reproductive counseling change, subspecialty consult initiation, medication change, procedure change, diet change, palliative care initiation, imaging change, and patient transfer to different facility, in a study aiming to identify Mendelian disorders in critically ill infants (45). For example, patient 38 was a six-year-old male with insulin-dependent diabetes, cataracts, and hyperglycemia, who did not fit the classic patterns of type 1 diabetes mellitus, maturity onset diabetes of the young (MODY), non-ketotic hyperosmolar syndrome, or neonatal diabetes. Upon exome sequencing, he was found to be heterozygous for a *de novo* missense mutation in the insulin gene (*INS*) which is associated with permanent neonatal diabetes mellitus or type 1b diabetes mellitus. Identifying the exact genetic cause of a patient’s diabetes is important as it directs future monitoring and treatment. Testing for monogenic diabetes is important and should be considered in any pediatric patient with antibody negative diabetes. In monogenic diabetes (*GCK* mutations excluded), monitoring for long-term complications such as retinopathy, neuropathy, and proteinuria should be considered at diagnosis given the long prodrome of unrecognized hyperglycemia. As science advances, patient 38 may be an ideal candidate for beta cell transplantation as the risk for islet cell antibody-mediated attack would be negligible. In addition to changing imaging and/or medication and initiation of subspecialty consults, a broader meaning of altered management was used in other exome positive cases which included receiving genetic counseling and ending the diagnostic odyssey (Table 2).

### Secondary findings

In addition to the diagnostic findings, 36 of 40 patients chose to receive secondary finding results. Three of the 36 patients had reported medically actionable secondary findings in a total of three genes (Table 2; Figure 1). Genes *MYL2*, *FBN1*, and *BRCA2* were among the medically actionable genes recently recommended for reporting by the ACMG (25). For example, Patient 23 was 2 years old at the time of testing. He presented with hypogammaglobulinemia, recurrent infections, fine motor and speech delay, and feeding problems. Dysmorphic features included macrocephaly, prominent forehead, deep set eyes, thin upper lip, long digits, and persistent fetal finger pads. Although WES did not identify the cause of the primary clinical features, it importantly identified a *de novo FBN1* mutation which gave the patient a secondary genetic diagnosis of Marfan syndrome. Upon the time of results return, he had developed hypermobility, gait abnormality, and abnormal posture. He was referred for cardiologic and ophthalmologic evaluations and for subsequent management for Marfan syndrome.

### Cost analysis

**Figure 2:**
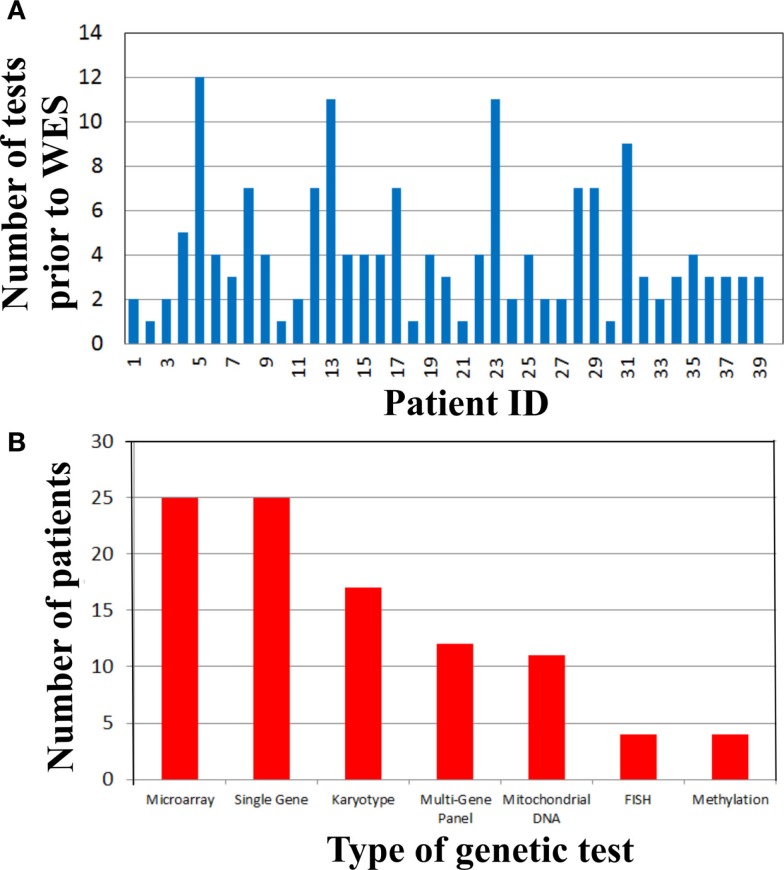
**Genetic testing cost analysis of cohort prior to whole exome sequencing**. **(A)** Number of genetic tests performed before whole exome sequencing per patient. **(B)** Types of genetic tests completed in the cohort prior to whole exome sequencing.

To address the cost of genetic testing prior to WES and consequently the potential cost-effectiveness of WES as a single test, we examined the number and type of genetic tests performed in our cohort prior to WES (Figure 2). Strikingly, there were 19 (48%) patients who had at least four genetic tests prior to WES (Figure 2A). From these 19 patients, three patients had a large number of genetic tests (>10 tests). The mean genetic test number for the cohort was ~4. Interestingly, microarray (63%) and single gene sequencing (63%) tests were the most frequently performed in this cohort, followed by karyotype and multi-gene panel sequencing. Therefore, the cost of genetic testing before WES is significant.

## Discussion

On applying WES to the diagnoses of 40 unselected consecutive patients, we observed an overall molecular diagnostic yield of 30%, which is slightly higher than the positive rates of reported clinical exome tests of ~25% (Figure 1) (18, 19). This may be the result of different categories of presentation, sample bias, sample size, employment of a dual analysis pipeline, and phenotyping. The inclusion of dual analysis pipelines (NGP and GGHP) allowed the test to be more sensitive and in some cases showed that variants not captured by one pipeline were indeed detected by the other. Alternatively, because patients with multiple congenital anomalies had the highest diagnostic yield of any group (50%) (Figure 1; Table 2), referring physicians may have selected patients most likely to have a genetic etiology based on striking phenotypic attributes. Moreover, WES provided a diagnosis to 17% of patients with both neurological disorders and immunodeficiencies. About 8% of patients had both multiple congenital anomalies and either immunodeficiency or neurological disorders. Our results suggest that these three groups of patients are good candidates for testing with WES.

The utility of WES is higher than karyotype analysis (5–15%), chromosomal microarray analysis (15–20%), and may be comparable to disease-specific gene panels (46–48). The diagnostic yield of single gene tests vary from 0 to 64% depending on phenotype specificity and availability of complementary diagnostic tests (49). Similarly, previous studies that have employed next-generation sequencing approaches have shown varying positive rates. NGS panel positive rates for muscular dystrophy, sporadic intellectual disability, severe intellectual disability, immunodeficiencies, and retinitis pigmentosa have been reported to be 41, 31, 13, 15, and 82%, respectively (19, 49, 50). Thus, the WES positive rate is comparable to many other genetic tests that are considered part of the routine diagnostic workup.

Time-consuming, extensive and costly clinical diagnostic workups were performed before ordering the clinical WES test. This workup included a significant number of genetic tests (Figure 2). In fact, there were 19 (48%) patients who had at least four genetic tests prior to WES (Figure 2A) and microarray and single gene sequencing were the most frequently performed genetic tests (Figure 2B). These data demonstrated that costly genetic testing was performed prior to WES and in that in some cases (>10 genetic tests) the combination of genetic tests was even more expensive than WES itself. One explanation for this is that older technologies were employed in the diagnostic workup of some of these patients because those were the only tests available at that time. The cost of reaching a diagnosis became astronomical when the clinical work of non-genetic testing was added to the entire diagnostic testing equation. For example, patient 29 had chromosome and microarray analyses, mitochondrial studies, very long chain fatty acid study, brain MRI, spine MRI, muscle biopsy, and an EEG. He had consultations with a myriad of specialists including geneticists, ENT, orthopedics, and neurologists. This patient has a mutation in *ASXL3*, which is associated with the newly recognized Bainbridge-Ropers syndrome that would not have been identified by conventional genetic testing because it was not available at the time of diagnosis. Currently, one clinical laboratory started offering this gene as a clinical test in the United States in March of 2015. In some instances, WES may be the most cost-effective way to reach a diagnosis and guide appropriate management by significantly reducing the time to diagnosis and cost of testing, if it is implemented at an earlier time in the diagnostic workup of complex genetic cases. To improve clinical and cost outcomes more broadly, diagnostic algorithms that include WES testing need to be created and implemented in the near future.

WES testing is more comprehensive than other single gene and panel tests, but it is laborious and therefore uses expensive human resources. However, it is difficult to quantify these costs because analysis pipelines are constantly evolving and making the process faster by utilizing a growing powerful internal and public control datasets as an example. Caution should be taken when interpreting cost analysis numbers because exome sequencing and its complementary analysis is likely to continue to decrease in the near future, perhaps with the introduction of new sequencing technologies and clever algorithms that will find candidate variants quickly.

Even though microarrays, karyotyping, FISH, and methylation are non-sequencing assays, they were included in the cost analysis because there are known examples of overlapping clinical features caused by sequence mutations found by WES or other mutation types detected by the aforementioned techniques. The typical indications for microarray deletion/duplication analysis (SNP microarray) include developmental delay, intellectual disability, congenital anomalies, and dysmorphic features. These clinical characteristics were observed in several of our WES cases and microarray testing was pertinent and ruled out large deletion/duplication events and thus WES was the next step of the diagnostic workup because there are single gene mutations that are responsible for similar clinical characteristics (Table 1). In addition, another potential use of SNP microarray analysis is to identify loss of heterozygosity (LOH) regions in consanguineous cases. This information in conjunction with WES testing may aid in identifying the regions to be examined for homozygous variants. However, this information is not crucial because WES analysis includes a homozygous genotyping model and would identify candidate homozygous variants. Choosing to do other genetic testing prior to WES analysis and any other genetic testing permutations depend on specific clinical scenarios, thus, inclusion or exclusion of non-sequencing tests in cost analyses must be taken with caution.

Interestingly, 33% of our 12 patients with positive results were based on disease–gene discoveries made within the past 2 years (Table 2). Specifically, four patients, including those with mutations in *NFKB2*, *PAX1*, *CTPS1*, and *ASXL3*, would not have received a specific clinical diagnose if their exome data had been analyzed earlier than 2 years ago, prior to published works describing the gene–disease associations (31, 51–54). During this explosion of genomic data, it is likely that we will see the literature and databases populated with more cases that will aid in the interpretation of clinical exome data. There is also the possibility of an evolving phenotype that might at some point alter or add to the diagnosis in some patients (19). This new information may aid in the interpretation of VUCS variant calls (Table 3). However, in other cases extensive future research, which includes RNA-expression studies, needs to be performed to clarify the significance of VUCS variants.

Variants in multiple genes with described clinical phenotypes were identified in several patients. In a patient with common variable immunodeficiency (CVID)-like symptoms, Lindsley et al. addressed the functional consequences of mutations in *NFKB2* [c.2598_2599insT (p.A867fs)] and *TNFRSF13B* [c.706G > T (p.E236*)] (Tables 2 and 3) (26). They reported the comprehensive immune evaluation of patient 4 and provide evidence that aberrant NFKB2 signaling not only causes humoral immune deficiency, but also interferes with the TCR-mediated proliferation of T cells. These observations expand the known phenotype associated with NFKB2 mutations. Unexpectedly, Western blot analysis of lysate from fresh, unstimulated PBMCs from the patient and father revealed a normal TNFRSF13B full-length protein. Yang et al. described multiple “hits” in four patients in their initial report and in a recent larger study, consisting of a cohort of 2000 consecutive exome cases, the same group reported a 4.6% positive rate for patients with multiple affected genes (19, 20). For atypical phenotypes, WES can expand the differential to include conditions that would otherwise not be considered. Additionally, WES is bound to expand the phenotype spectrum by the characterization of more complex and atypical cases.

The medical management of patients was altered after obtaining a positive exome test. Willig et al. have defined medical management in a broader sense in a study aiming to identify Mendelian disorders in critically ill infants (45). The definition of medical management includes usefulness of genomic sequencing, return of results before discharge or death, genetic or reproductive counseling change, subspecialty consult initiation, medication change, procedure change, diet change, palliative care initiation, imaging change, and patient transfer to different facility. From the traditional definition of medical management perspective, identifying the exact genetic cause of a patient 38’s antibody negative diabetes was important to direct future monitoring and treatment. Monitoring retinopathy, neuropathy, and proteinuria were considered at diagnosis (Table 2). Similarly, WES allowed change of management for patient 23 by being referred for cardiologic and ophthalmologic evaluations after the secondary finding of Marfan syndrome. In the broader sense of medical management, positive WES results altered the management of corresponding patients because they received genetic counseling, ended the diagnostic odyssey, changed imaging and/or medication, and initiated subspecialty consults to mention a few points.

Negative results for cases in which a diagnosis was not reached may be due to the lack of understanding of the exome, technical limitations, and patient selection bias. There are a large number of coding genes that have not been associated with human diseases and much needed genetic and functional studies should be done to elucidate their function. It is possible that some of these genes of unknown clinical significance (GUCS) may help explain the clinical features in several of our negative cases in the future. Thus, it is important to perform re-analysis on exome data annually to interrogate for newly discovered genes with human disease associations. Moreover, mutations may be located in non-coding regions, such as regulatory or deep intronic regions, that cannot be detected by WES. Moreover, mutations may be located in low coverage coding regions. Due to WES being a hybridization-based assay, the presence of multiple pseudogenes, homologous regions, or repetitive regions poses a technical challenge that obscures the presence of variants (55). In addition, large deletion/duplication mutations, complex rearrangements, trinucleotide repeats, and imprinting changes may be missed by WES and may represent a significant portion of mutations in the negative cases. It is possible that the clinical presentations of some of the patients could be explained by polygenic effects (complex disease) or a non-genetic etiology.

In conclusion, the use of WES to analyze 40 consecutive clinical cases yielded a diagnosis in 30% of these cases, which demonstrates the utility of this technology as a diagnostic test for pediatric patients with a wide variety of disease presentations. Positive WES results allowed clinicians to complete the genetic workup, end the diagnostic odyssey and provide appropriate medical management and more informative genetic counseling to families. Importantly, a number of novel mutations are being reported here. The cost-effectiveness of WES testing is evident by the reduction of time to diagnosis and cost of other testing and in some cases WES may be warranted as a first-tier test. Although there are technical challenges with NGS, WES provides a unique glimpse into the complexity of genetic disorders as well as the challenges in diagnosing them. However, healthcare system integration and routine adoption of WES need more careful consideration and future research.

## Author Contributions

Drs. CV and KZ conceptualized and initiated the study, collected, analyzed, and interpreted the data, wrote and reviewed the manuscript; Mrs. AH, AM, TT, Mses. JJ and JH, and Drs. YQ, MK, KK, and JH developed the WES test and reviewed the manuscript; Drs. CW, SI, FZ, HM, XL, LW, and AB performed the data analyses and reviewed the manuscript; Mses. KC and KW, and Drs. TB, ES, DN, and RH provided patient’s clinical information and reviewed the manuscript. All authors approved the final manuscript as submitted and agree to be accountable for all aspects of the work.

## Conflict of Interest Statement

The authors declare that the research was conducted in the absence of any commercial or financial relationships that could be construed as a potential conflict of interest.

## Supplementary Material

The Supplementary Material for this article can be found online at http://journal.frontiersin.org/article/10.3389/fped.2015.00067

## Abbreviations

ACMG, American College of Medical Genetics; CAP, College of American Pathologists; CASAVA, consensus assessment of sequence and variation; IRB, Institutional Review Board; MODY, maturity onset diabetes of the young; VUCS, variant of unknown clinical significance; WES, whole exome sequencing.
