# Supplementary material for: Clinical Impact and Cost-Effectiveness of Whole Exome Sequencing as a Diagnostic Tool: A Pediatric Center’s Experience
Source: Front Pediatr. 2015 Aug 3;3:67. doi: 10.3389/fped.2015.00067 (PMC4522872; doi:10.3389/fped.2015.00067)
Supplement: Supplementary file 1 [file Table_1.PDF]

Supplemental Table 1.

Case ID	Number of Variants		Quality parameters		
	Prior to filtering	After filtering	Average coverage per family#	Percentage at 10X coverage per family	Percentage at 20X coverage per family
1	34334	7502	155.67	96.91	95.76
2	33306	6865	140.00	96.91	95.77
3	25108	4120	132.67	96.78	95.47
4	23852	3000	152.00	96.83	95.44
5	34288	3682	128.00	96.96	95.64
6	25546	1763	127.50	97.05	95.72
7	25553	1404	107.00	96.88	95.29
8	34772	4117	134.67	96.57	95.14
9	25113	1075	131.67	96.65	95.37
10	24981	1239	123.00	96.65	95.09
11	33674	1072	131.33	96.86	95.60
12	25719	1374	129.33	96.94	95.67
13	33547	1210	138.67	96.79	95.45
14	35758	649	118.75	96.77	95.26
15	33891	1615	185.67	97.52	96.56
16*	17603	1523	82.00	96.47	93.95
17	34117	1470	128.33	96.87	95.53
18	36147	1139	104.50	96.48	94.30
19	34699	656	132.75	97.02	95.69
20	36890	1621	126.20	96.90	95.30
21	34594	1829	152.67	97.36	96.32
22	34823	1535	131.00	97.33	96.12
23	35143	871	104.50	96.00	94.12
24*	23922	3411	80.00	95.54	93.27
25	34555	1498	148.67	96.86	95.72
26	34741	1754	150.33	96.89	95.63
27	33144	1571	102.00	96.26	94.71
28	34109	1913	113.00	96.35	94.81

29	34489	1513	102.67	96.55	94.80
30	33971	1566	108.67	96.82	95.53
31	34526	1968	114.33	97.34	95.91
32	34243	1566	111.00	97.11	95.52
33	36683	1779	99.33	96.91	94.99
34	34156	1683	193.00	97.85	97.01
35	34020	1603	135.00	97.61	96.53
36	36369	1715	88.33	96.53	94.72
37	33002	1781	109.33	96.78	95.28
38	33666	1582	92.67	96.74	95.04
39	33619	1479	127.00	96.99	95.65
40	34164	1508	157.00	97.38	96.35
Average	32171	2031	125.76	96.85	95.40
Standard Deviation	4547	816	24.78	0.42	0.72

*proband only
